# Precision Health Optimization Program in the Management of Advanced Parkinson’s Disease: A Case Series

**DOI:** 10.7759/cureus.101146

**Published:** 2026-01-09

**Authors:** G Niraj, N Charan, Shruti Niraj, Varsha B Karanth, Lakshmanan Prakash

**Affiliations:** 1 Pain Medicine and Anesthesia, Sri Madhusudan Sai Institute of Medical Sciences and Research, Chikkaballapur, IND; 2 Pain Medicine and Palliative Care, Sri Madhusudan Sai Institute of Medical Sciences and Research, Chikkaballapur, IND; 3 Psychology and Palliative Care, Sri Madhusudan Sai Institute of Medical Sciences and Research, Chikkaballapur, IND; 4 Psychiatry, Sri Madhusudan Sai Institute of Medical Sciences and Research, Chikkaballapur, IND; 5 Orthopedics, Institute of Special Orthopaedics, Palakkad, IND

**Keywords:** autonomic dysfunction, blood ozone therapy, mitochondrial dysfunction, molecular hydrogen therapy, niraj prakash program, nutraceutical infusion, oxidative stress, parkinson’s disease, stellate ganglion block, sympathetic overactivity

## Abstract

Parkinson’s disease (PD) is reported to be the fastest-growing neurodegenerative condition with significant management challenges. Neurobiology is linked to mitochondrial dysfunction, oxidative stress, autonomic imbalance, and neuroinflammation. There is progressive propagation of alpha-synuclein protein in neural networks and dopamine deficiency in the nigrostriatal pathway. Levodopa remains the gold standard agent in its management. However, the non-motor symptoms that significantly compromise quality of life remain largely unresponsive to levodopa. Currently, there are no interventions that can modify disease progression. Following a comprehensive assessment by an interdisciplinary team, three patients with advanced idiopathic PD were treated with a precision health optimization program. The program included dual stellate ganglion block, nutraceutical infusions, and extracorporeal blood ozone therapy. The program targets the underlying autonomic imbalance, oxidative stress, neural inflammation, and mitochondrial dysfunction. Validated outcome measures were completed at baseline and at the 12-week review. At the 12-week review following the program, all three patients reported a reduction in disease severity with an improvement in quality of life. The innovative precision health optimization program could have a role in the management of advanced PD. Limitations include a small patient cohort, the absence of a control arm, and potential placebo effects.

## Introduction

Parkinson’s disease (PD) is a progressive neurodegenerative condition that is characterized by motor deficits and non-motor symptoms. PD is classified into genetic and sporadic. Sporadic PD accounts for a substantial proportion of patients, and genetic contributions exist along a spectrum. Diagnosis is based on three core motor symptoms: bradykinesia, rigidity, and resting tremor [1]. Additional motor symptoms include speech difficulties, hypophonia, muscle dystrophy, pain, decline in facial expression, postural deformities, gait freezing, dyskinesia, and instability. Non-motor symptoms occur throughout the disease course, from early to late stages, and have a pronounced negative impact on quality of life [2]. Levodopa remains the gold standard agent. However, it is often incompletely responsive in managing posture and gait imbalance, speech difficulty, freezing, autonomic imbalance, cognitive disorders, anxiety, and sleep impairment [1,2]. Additional therapeutic agents include monoamine oxidase inhibitors, catechol-O-methyltransferase (COMT) inhibitors, and N-methyl-D-aspartate receptor antagonists [1]. In advanced PD, the core motor and non-motor symptoms, as well as motor complications, can become increasingly unresponsive to standard dopaminergic medications. In this scenario, the surgical option includes deep-brain stimulation [1]. When advanced PD becomes unresponsive to treatment in elderly patients with substantial co-morbidities, clinicians have very few therapeutic avenues available. We present the successful management of advanced PD with a precision health optimization program in three elderly patients. Following a comprehensive assessment by an interdisciplinary team, the patients were treated with dual stellate ganglion block (PD), nutraceutical infusions, and extracorporeal blood ozone therapy (EBOT). These interventions target dysautonomia, oxidative stress, mitochondrial dysfunction, and neural inflammation that are reported in the neurobiology of PD [3-5].

## Case presentation

Three patients with advanced idiopathic PD presented to the Pain Medicine and Palliative Care clinic at Sri Madhusudan Sai Institute of Medical Sciences and Research, a tertiary care medical college located in southern India. The patients were on a holiday and were visiting a nearby spiritual retreat. Patients underwent an initial neurologic review upon presentation to the hospital. The interdisciplinary team comprised a palliative care physician, a clinical psychologist, a psychiatrist, and a pain medicine physician. The patients underwent a comprehensive assessment by the IDT. Both patient-reported and clinician-reported validated outcome forms were completed at baseline and at the 12-week review. These included the Unified Parkinson’s Disease Rating Scale (Original UPDRS), the Generalized Anxiety Scale (GAD-7), and the Edmonton Symptom Assessment Scale revised (ESAS-r), which were completed by clinicians trained to administer these questionnaires. Investigations included echocardiogram, glucose-6-phosphate dehydrogenase (G6PD) enzyme assay, liver function and renal function tests, erythrocyte sedimentation rate, and full blood count.

Patients were provided an information sheet detailing the program and the rationale for offering it. The Precision Health Optimization program is part of the standard care offered to all patients with severe chronic health disorders attending the palliative care clinic at our center. Informed written consent was obtained. Additional written consent was obtained for their de-identified data to be used for publication in a peer-reviewed journal.

A separate informed written consent was obtained for receiving EBOT, which was defined as a complementary treatment in palliative care. Currently, ozone therapy is offered within complementary therapy settings in India [6].

Case 1

An 82-year-old female patient, a resident of the United Kingdom (UK), presented to the IDT clinic with a 10-year history of PD (modified Hoehn and Yahr Stage 5). Six months prior to presentation, she had developed decompensated heart failure with preserved ejection fraction requiring protracted hospital admission. At presentation, she was wheelchair bound and exhibited impaired facial expression, resting tremor, significant insomnia, shortness of breath, drooling, sweating, and severe anxiety. Additionally, she reported low mood and widespread pain, which was poorly controlled on a combination of opioid and non-opioid medications. Medications included co-beneldopa 125 mg QID (levodopa equivalent daily dose = 400 mg), bumetanide 2 mg, tramadol 100 mg, gabapentin 200 mg, and paracetamol 3 g. The patient was unable to tolerate a trial of transdermal rotigotine 2 mg/24 hours and COMT inhibitors in the UK.

On examination, she was unable to rise from the wheelchair or walk unaided. There was pitting edema up to the knees and postural hypotension. Blood pressure readings were 114/69 mm Hg in the supine position and 84/55 mm Hg on standing for three minutes. As per a clinic letter, an echocardiogram performed in the UK showed heart failure with preserved ejection fraction.

She underwent the precision health optimization program over a five-week period. During this period, she did not receive any other therapy, including physical therapy. At the 12-week review, she was able to walk unaided, rise independently from a chair, and smile. She reported minimal anxiety and improved mood and sleep pattern. Additionally, she reported a complete absence of pain, drooling, nocturnal shortness of breath, and sweating.

Examination revealed minimal pedal edema. There was significant improvement in postural hypotension (supine blood pressure (136/63 mm Hg) and on standing for three minutes (118/69 mm Hg)). A repeat echocardiogram showed normal biventricular function and size, an ejection fraction of 60%, an E/e ratio of 11.2, a left atrial size of 38 mm, and grade 1 left ventricular diastolic dysfunction. Baseline and 12-week outcomes were collected by a trained clinician during the on-period and are reported in Table 1. She was able to discontinue all medications except co-beneldopa.

**Table 1:** Patient characteristics and outcomes PD: Parkinson’s disease, UPDRS: Unified Parkinson’s Disease Rating Scale, GAD-7: Generalized Anxiety Scale, ESAS-r: Edmonton Symptom Assessment Scale-revised

	Age (year, gender	Baseline PD severity (modified Hoehn and Yahr staging)	12-week PD severity (modified Hoehn and Yahr staging)	Baseline original UPDRS	12-week original UPDRS	Baseline self-reported unassisted walking distance (yards)	12-week self-reported unassisted walking distance (yards)	Baseline GAD-7 score	12-week GAD-7 score	Baseline ESAS-r score	12-week ESAS-r score
Case 1	82, F	5	4	101	32	5	50	20	11	73	21
Case 2	88, M	4	3	60	32	30	500	14	7	33	13
Case 3	79, M	4	3	90	36	20	500	16	9	53	27

Case 2

An 88-year-old male resident of the United Arab Emirates (UAE) presented with a 10-year history of PD (modified Hoehn and Yahr stage 4). He was receiving treatment in the UAE. During his current visit to India, he suffered an acute cerebral infarction due to a thrombosis of the left middle cerebral artery. He was also diagnosed with carcinoma of the bladder. Although he recovered power in all limbs within three weeks of the stroke, he reported cognitive decline, unsteady gait, and significant fatigue, which worsened following the stroke. He had completed a three-week course of intensive physiotherapy. The patient’s self-reported walking distance reduced from 100 yards (pre-stroke) to 30 yards with a walker. Additional symptoms included impaired facial expression, resting tremor (left > right hand), significant rigidity, bradykinesia, and low mood. Medications included aspirin 75 mg, bisoprolol 1.25 mg, amlodipine 2.5 mg, levodopa 300 mg, and carbidopa 75 mg. He was unable to tolerate an increase in the dose of levodopa, as well as a trial with COMT inhibitors.

At the 12-week review following the program, the patient reported significant improvement in fatigue, sleep, mood, and memory. The self-reported walking distance increased from 30 yards to 500 yards. Outcomes are reported in Table 1.

Case 3

A 79-year-old male presented to the IDT with a six-year history of PD (modified Hoehn and Yahr rating stage 4). He was receiving treatment in Italy. His symptoms worsened significantly following general anesthesia for an inguinal hernia repair 12 months ago. Postoperative recovery was complicated by prolonged delirium and immobilization.

Comorbid conditions included hypertension, severe anxiety, and insomnia. There was significant difficulty in falling asleep, multiple awakenings due to pain in the lower limbs, and waking up unrefreshed. He could manage only one hour of sleep at night. Additionally, he reported significant rigidity, low mood, resting tremor, constipation, nocturia, and urgency. Medications included levodopa 400 mg, carbidopa 100 mg, furosemide 50 mg, and spironolactone 37 mg.

On examination, he was unable to stand up from the wheelchair unaided. He could manage 20 yards with a walker. There was pitting edema till the mid-thigh. The echocardiogram showed mild left ventricular diastolic dysfunction and an ejection fraction of 60%.

At the 12-week review following the program, he was able to walk independently for 300 yards and arise from a supine position unaided. He reported minimal anxiety and an improved mood with reduced constipation, nocturia, and urgency. He slept for five hours at night and reported no pain in the lower limbs. Examination revealed minimal pedal edema. His family noted substantial improvement in facial rigidity. Outcome scores are reported in Table 1.

Precision health optimization program (Niraj Prakash program)

Week 1

On Day 1, a comprehensive IDT assessment was conducted. On Days 2 and 3, a dual, two-level SGB was performed. The procedure was performed as an outpatient procedure under cardiovascular monitoring. Technical details of ultrasound-guided SGB have been previously reported [7]. The injectate consisted of 5 ml of 0.25% bupivacaine, 1 ml of 2% lidocaine, 20 mg of depot methylprednisolone, and 6 ml of distilled water. The right-sided block (total volume 12 ml) was performed first at the C6 (7 ml) and C4 (5 ml) levels. The left-sided blocks were performed within 96 hours. Depot steroid was added to enhance the block's durability [7].

On Day 4, the patient underwent EBOT. EBOT was offered only if the patient had normal G6PD levels. It was performed in the pain medicine intervention room under aseptic precautions and cardiovascular monitoring. A large-gauge cannula was inserted into a suitable vein and connected to an infusion system primed with heparinized saline (7500 units of heparin diluted in 500 mL saline). The infusion system was connected to a mechanical pump that enabled blood to be drawn out of the patient at a rate of 25-35 ml/minute and pumped into a special, flint-free, plastic-free, renal filter-free glass bottle. The collected blood was ozonized at 90 mcg/ml using a Mark 8 IAOS Medical Ozone Generator (Chennai, TN, India). The ozonized blood was transfused back into the patient via a second intravenous cannula. The closed-loop system continued until 1-1.2 L of blood was ozonized and re-transfused into the patient. The procedure lasted 30-45 minutes. A peripheral smear examination was performed pre- and post-therapy to rule out hemolysis following EBOT.

On Day 6, a nutraceutical infusion was administered. The infusion comprised high-dose vitamin C (0.5 g/kg), 1.2 g of glutathione, and 1 g of magnesium sulfate, mixed in 250 ml of normal saline, infused intravenously over 45 minutes. Hydrogen gas (66% H₂ and 33% O₂) was bubbled in the infused saline bottle (Mark 3, IAOS Hydrogen Generator, Chennai, TN, India). Key precautions were taken, including use by trained personnel, adequate ventilation, elimination of ignition sources, and use of compatible material and equipment.

Weeks 2-4

On Day 1, the patient underwent EBOT. Nutraceutical infusions were administered on Days 3 and 6. In total, the patients received four sessions of EBOT and seven sessions of nutraceutical infusion. Following EBOT, peripheral blood smears showed no evidence of hemolysis in any of the three patients. No complications or adverse events were observed throughout the program.

## Discussion

We present the successful management of three advanced PD patients with an innovative precision health optimization program. Upon completion of the program, all three patients’ walking distance, sleep patterns, and rigidity improved substantially, while mental health stabilized. Elderly patients with advanced PD have limited treatment options. The presence of major comorbidities precludes surgical intervention. In addition to severe rigidity, non-motor symptoms result in severe dysfunction and impaired quality of life. Currently, no therapy has been shown to slow down or arrest the progression of PD [2]. To the best of our knowledge, this is one of the first reports to describe the benefits of a precision health optimization program in managing advanced PD.

The pathophysiology of PD is characterized by the presence and progressive propagation of the alpha-synuclein (α-syn) protein and a loss of dopaminergic neurons [3]. Neurobiological factors implicated in PD include oxidative stress, mitochondrial dysfunction, and neuroinflammation [4]. These patients often present with dysautonomia. There is emerging evidence that a dysfunctional autonomic nervous system is a potential route by which PD pathology spreads to and from the central nervous system [3]. Dysautonomia often precedes motor symptoms and can affect the quality of life [5]. SGB targets the underlying autonomic imbalance by dampening sympathetic overactivity.

Additionally, SGB also enhances parasympathetic activity and alleviates stress response [8,9]. In this cohort, dual two-level SGB produced a substantial reduction in anxiety [7]. SGB also improved sleep patterns, reduced excessive sweating, and mitigated orthostatic hypotension.

High-dose vitamin C maintains the integrity of mitochondrial cellular membranes and thereby prevents oxidative stress [10]. Vitamin C also prevents oxidation-induced DNA mutations and lipid peroxidation and induces the repair of oxidized amino acids [11]. There is early evidence that nutraceutical infusion containing vitamin C could benefit patients with PD [12]. Additionally, vitamin C has been reported to enhance levodopa absorption in elderly patients [13]. Although clinical studies on the efficacy of dietary vitamin C in PD have been disappointing, the effectiveness of high-dose intravenous vitamin C remains to be explored. All three patients in this cohort reported a significant reduction in cogwheel rigidity and freezing. This could likely be due to enhanced levodopa absorption and mitigation of oxidative stress.

Ozone therapy is reported to enhance cerebral microcirculation and facilitate neuronal energy production [14-16]. Blood ozone therapy causes a transient oxidative stress followed by a sustained antioxidant effect. The protective effects manifest through enhanced nuclear factor erythroid 2-related factor 2 activity, orchestrating up-regulation of key protective enzymes while suppressing excessive nuclear factor kappa B-mediated inflammation. Ozone therapy activates antioxidant, anti-apoptotic, and pro-autophagy pathways [14-16]. Current evidence demonstrates consistent protective effects across various organ systems [17]. In the present cohort, there was a substantial reduction in widespread pain with marked improvement in cognition and walking distance. These effects may be associated with EBOT. The authors acknowledge the possibility of placebo effects, regression to the mean, neutral fluctuations, and concurrent changes in care.

Hydrogen gas has unique antioxidant properties, as it selectively counteracts deleterious reactive oxygen species and has a good safety profile [18]. Animal studies have reported that molecular hydrogen functions as a highly effective antioxidant therapy in PD [19]. However, inhaling hydrogen gas has not shown benefits in PD [20]. The potential benefits of intravenous infusion of hydrogen gas bubbled in normal saline have not been formally evaluated.

It was interesting to observe an improvement in cardiac function in Cases 1 and 3. In addition to a significant improvement in pedal edema, there was demonstrable improvement in sweating and postural hypotension in Case 1.

The authors acknowledge significant limitations, including an observational report of a small cohort of patients (n = 3) with heterogeneous presentations, a short follow-up period, no blinded assessment, and no control arm. Additionally, the improvement in function could have been coincidental, due to a placebo effect and/or regression to the mean. However, there is a pressing need for innovative paradigms to decelerate or halt the progression of PD. The psychosocial impact of advanced PD is substantial. Since various etiological factors are involved in the neurobiology of PD, management should be multi-pronged. Thus, the innovative program targets dysautonomia, mitochondrial dysfunction, oxidative stress, and neuroinflammation, thereby mitigating the pathophysiological factors implicated in PD.

## Conclusions

Disease modification and enhancing symptom management remain key research priorities in elderly patients with advanced PD. The innovative precision health optimization program targets key neurobiological pathways linked to PD pathogenesis. It could have a role in the management of advanced PD. Definitive studies are warranted.
